# Perturbation‐induced responses improved seizure forecasting in epileptic rats

**DOI:** 10.1002/epi.70196

**Published:** 2026-03-13

**Authors:** Wei‐Chih Chang, Jack Lin, Warwick Cheung, Alan Lai, Mark J. Cook, David B. Grayden, William C. Stacey

**Affiliations:** ^1^ Department of Molecular & Integrative Physiology University of Michigan Ann Arbor Michigan USA; ^2^ Department of Neurology University of Michigan Ann Arbor Michigan USA; ^3^ Department of Medicine St. Vincent's Hospital Melbourne, University of Melbourne Melbourne Victoria Australia; ^4^ Graeme Clark Institute for Biomedical Engineering University of Melbourne Melbourne Victoria Australia; ^5^ Department of Clinical Neurosciences St Vincent's Hospital Melbourne Melbourne Victoria Australia; ^6^ Department of Biomedical Engineering University of Melbourne Melbourne Victoria Australia; ^7^ Department of Biomedical Engineering, Biointerfaces Institute University of Michigan Ann Arbor Michigan USA; ^8^ Division of Neurology VA Ann Arbor Healthcare System Ann Arbor Michigan USA

**Keywords:** brain stimulation, critical slowing down, critical transition, perturbation, seizure forecasting and prediction

## Abstract

**Objective:**

The unpredictability of seizures is one of the most challenging aspects of uncontrolled epilepsy for patients. Prior work forecasting seizure risk has measured changes in passive intracranial electroencephalographic (EEG) signals, but currently, there are no such clinical devices available. Based upon dynamical theory, we hypothesized that the response of the brain to perturbing stimulation provides a robust measurement of seizure risk that outperforms the results from passive EEG.

**Methods:**

To test the hypothesis, we performed more than 8 weeks of periodic electrical stimulation and continuous EEG recordings in epileptic rats induced by intrahippocampal injection of tetanus toxin, in which seizures started spontaneously.

**Results:**

Using the perturbation‐evoked responses as a predictive biomarker of seizure risk, we built a preictal detection system that had excellent accuracy (area under the receiver operating characteristic curve > .95) at distinguishing the preictal from the interictal states. In comparison, a similar preictal detection system that used only passive features from the same experimental animals was unable to identify the preictal state better than chance.

**Significance:**

Our results advocate for perturbation to be used for seizure prediction purposes, which could improve the efficacy of seizure forecasting when applied clinically.

**Key points**

- We tested whether electrical perturbation‐induced responses could be used to identify the preictal state in epileptic rats.
- A multivariable logistic regression of five features of the evoked response accurately discriminated the preictal from interictal state, with area under the receiver operating characteristic curve up to .95.
- A similar approach using features from passive (i.e., nonperturbed) recordings failed to discriminate the preictal states.

## 1 INTRODUCTION

Epilepsy is a common brain disease defined by spontaneous and recurrent seizures that disrupt normal brain activity. Seizures arise abruptly and cause physical injuries and social embarrassment to people with epilepsy. Modern treatments include medications, surgical resection, or surgical implantation of stimulation devices, but the risk of oncoming seizures continues to be a major source of patient anxiety^1^ and treatment failure. This gap in care has led to the search for strategies that would measure seizure risk over time and potentially “forecast” oncoming seizures.^2^, ^3^

After decades of research into seizure prediction or forecasting,^4^, ^5^ evidence suggests the onsets of seizures are not totally stochastic, but seizures predominantly happen at a particular hour(s), day(s), or month(s).^6^, ^7^ There have also been decades of research showing that many seizures are preceded by measurable electroencephalographic (EEG) changes.^4^, ^8^, ^9^ Human trials demonstrated that forecasting seizures in individual patients requires patient‐specific tuning, but results have progressed only modestly since the first trial.^10^ One potential weakness with prior strategies is that passive EEG recording may not be sensitive enough to identify the subtle changes that arise as seizures become more likely.^11^ The brain receives and generates countless signals at any given moment, so it is difficult to determine which are the salient signals related to seizure risk. In essence, the ictogenic neuronal network may not always be activated during passive recording. This idea has led some researchers to move beyond passive recordings and investigate how the brain responds to perturbations.

The onset and offset of seizures are critical transitions in brain state, moving from quiescence to bursting and back.^12^ These transitions have been well described in the physics literature using bifurcation theory,^13^ and have been shown to be present in human seizures.^14^, ^15^ That mathematical framework leads to important predictions about brain dynamics. One intriguing prediction is “critical slowing down,” a phenomenon in which the response to a perturbation becomes larger and slower when the system is closer to a bifurcation.^16^ This phenomenon is sometimes quantified as the “resilience.”^17^, ^18^ Several groups have demonstrated that humans have critical slowing when seizures are more likely.^18^, ^19^, ^20^, ^21^ However, there are two important limitations to that prior work. First, critical slowing is not the only possible response predicted by bifurcation theory; there are different bifurcation types that have different responses,^22^ which is not addressed by the prior work with perturbations. Recently, one group measured perturbations in inpatients for 24 h and found that even in this brief period the stimulus responses could forecast seizures better than passive EEG.^23^ But translation to use outside the hospital is still not possible, because current clinically approved devices are not designed to perform such protocols. To mitigate that limitation, one group treated interictal spikes as if they were perturbations in long‐term, passive EEG and found that there was evidence of critical slowing even with those limited perturbations that could be useful for seizure forecasting.^24^ Another alternative is to perform longer experiments in rodent models of epilepsy.^17^, ^18^

In this study, we applied repetitive, low‐amplitude stimulation over the entire course (several weeks) of the tetanus toxin (TeNT) model of epilepsy in rats, in which seizures manifest spontaneously and repeatedly and which is familiar to our group.^25^ One important aspect of this model is that the seizure bifurcations changed over the course, which corresponded to changes in the evoked responses.^25^ Here, we test the hypothesis that the perturbations would produce measurable responses that could be used to forecast the likelihood of upcoming seizures. Furthermore, we hypothesized that the evoked responses would perform better than passive data at forecasting seizures.

## 2 MATERIALS AND METHODS

**FIGURE 1:**
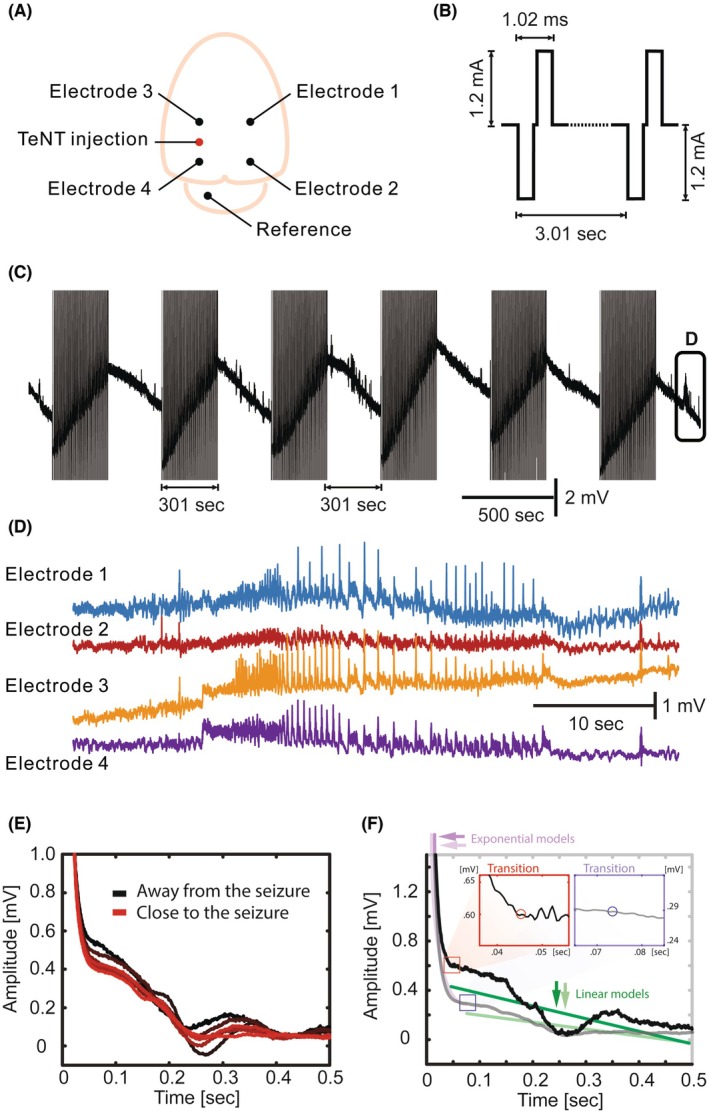
Experimental scheme and feature extraction. (A) A diagram indicates the electroencephalographic (EEG) electrodes flanking the hippocampi; tetanus toxin (TeNT) was injected into the left hippocampus of the rat. (B) Electrical perturbation comprises biphasic square pulses with the interstimulus interval equal to 3.01 s. (C) Trains of electrical stimuli periodically perturbed the selected 60‐min EEG (100 stimuli per stimulating train; intertrain interval = 301 s) and left half of the recording unperturbed; a seizure (D) happened during the unperturbed period when the electrical stimulation was off. The stimulating artifacts are truncated to reveal brain activity. (D) The seizure presented in all four EEG electrodes but initiated from the ipsilateral side to the TeNT injection side (Electrodes 3 and 4). (E) A diagram shows six time‐lapse evoked responses in the 2 h before a seizure from Electrode 4 (lapse = 20 min). The bright red trace was a 5‐min averaged evoked response just before the seizure onset; the darkness of traces decreases with lapses; thus, the black trace happened 105 min before the bright red one. (F) This diagram shows the initial analytic dissection for the evoked responses with or without “bumps.” We identified the transition point (red, with bumps; blue, no bump) between the fast and slow components, and the 500‐ms evoked response was divided into the exponential component and the linear component. The putative exponential (purple) and linear (green) fitting models are illustrated.

Detailed Materials and Methods are provided as Supporting Information. The animal ethics committee of St Vincent's Hospital, Melbourne approved the animal study, and we performed the experiments in accordance with the corresponding Australian code for animals for scientific use. This work is a secondary analysis of an experiment that was previously published.^25^ Six adult male Sprague–Dawley rats received TeNT (30 ng) intrahippocampally to induce epilepsy (Figure 1A) on Day −1; three male rats received only vehicle, serving as the control. Each animal was implanted with four epidural screw electrodes and a reference for almost nonstop, 24/7 recordings lasting for 2 months (Figure 1A). Consistent and periodic electrical stimuli using previous methods^26^, ^27^, ^28^, ^29^ (biphasic pulses, ±1.2 mA; 500 μs per monophase stimulus; 20‐μs gap; 100 stimuli per train; intertrain interval = 301 s; length = 1.02 ms) were delivered through Electrodes 3 and 4 to perturb the animals' neuronal network throughout the experiments (Figure 1B,C), ending after approximately 2 months (65.8 ± .8 days). The stimuli were delivered ipsilateral to the TeNT injection to the rats' brains, because that was the site of the primary seizure focus (Figure 1C). The recording and perturbation continued daily for ~23 h (82 663.1 ± 415.4 s) every day except for an hour of maintenance. This periodic on–off stimulating protocol started from the second day of TeNT injection and lasted until the end of every experiment. There was a direct current (DC) drift during the electrical perturbation (Figure 1C) that was carefully corrected during the analysis.

Spontaneous seizures were detected automatically by applying the published methods^25^; all detected seizures were verified manually by two physiologists (see Supporting Information). This study uses only the interictal data acquired from Electrode 4. Recordings were partitioned into alternating 301‐s perturbed sections and unperturbed sections based on the presence of electrical stimuli. In the perturbed sections, we extracted features from every stimulus‐evoked response (.5‐s discrete data) and acquired the means of the features in 10‐s rolling windows. A battery of features was chosen based upon signal characteristics (described later). We also drew features from the unperturbed sections in every 10‐s rolling window (10‐s continuous data). The nonictal data were divided into preictal periods (the 30 min before a seizure) and interictal periods (all data greater than 60 min before a seizure and 10 s after the preceding seizure). We used the receiver operating characteristic (ROC) and its area under the curve (AUC) to determine how well the features could discriminate between the interictal and preictal periods. Significance of the AUC values was determined by performing 500 permutations of the labels of interictal/preictal to generate a *p*‐value. The top five features from the perturbed and unperturbed sections, respectively, were selected and normalized to create a preictal detection system utilizing multivariable logistic regression. The normalized coefficients were used to determine which features were most important in the regression.

To control for time of day and housing effects, we compared data from each control rat (three total) with that of two experimental rats. We used the seizure time of an experimental rat to isolate the interictal data from a control rat, segmented the data into the interictal and preictal periods, and performed identical analyses on the data from the control animals. Although each rat was separately housed, pairing the data to the same time of day allowed us to assess external effects.

## 3 RESULTS

### 3.1 TeNT‐induced epilepsy in rats and electrical perturbations

As is common for this model of epilepsy, spontaneous seizures (Figure 1D) happened first on Day 5.0 ± 1.1 and remitted after Day 57.2 ± 3.3. Animals had 1642.3 ± 196.0 seizures during their lifetime. Seizures often formed clusters. A seizure cluster was not always followed by a prolonged seizure‐free period, but often the incidence of seizures reduced after clusters. Seizures lasted for 42.7 ± .2 s, and the average length of interictal periods was 1373.3 ± 22.9 s (median = 730 s). To investigate the possibility that the perturbing electrical pulses might trigger or suppress seizures, we evaluated the temporal relationship between seizures and the phases of electrical perturbations. There was no relationship between seizure occurrence and the timing of the stimuli (Supplementary S1).

### 3.2 Ability of individual perturbation features to discriminate preictal state

The first goal was to identify candidate features from the electrical perturbation‐evoked responses that are sensitive to the proximity of seizures. Additionally, we attempted to detect factors that might compromise the seizure forecast.

The averages of the perturbation‐evoked responses in 5‐min windows from Electrode 4 (that is ipsilateral, caudal to the TeNT injection; Figure 1A) suggested the evoked responses comprise fast and slow components. We noticed morphological changes happened approximately 100–500 ms after the stimuli during the slow component, which may be associated with the imminent seizures (Figure 1E). Therefore, we decided to divide the responses mathematically and to describe both components separately (Figure 1F), in addition to the power from the complete evoked responses. Altogether, we built a portfolio of 19 features (Table 1). We aimed to identify candidate features capable of signaling the proximity of seizures in a manner of tens of minutes; therefore, we monitored feature changes in 60‐min windows prior to seizures; individual preictal periods had to be at least 30 min long (348.2 ± 48.9 seizures per animal were included). We found quantitative changes or altered trends of features within the preictal 30 min (Supplementary S2), similar to the findings of preictal changes high‐frequency oscillations.^26^, ^27^ Thus, we chose 30 min as the potential preictal period. Because some of the features had some inconsistent changes between 30 and 60 min, we excluded that period as a buffer between preictal (0–30 min before seizure) and interictal periods (>60 min before seizure).

**TABLE 1:** List of features derived from the perturbed sections.

	Features	Definitions
	Transition point	
1	Latency to the transition point	Time at |d*V*/d*t*| ≈ 0
2	Amplitude at the transition point	Amplitude at |d*V*/d*t*| ≈ 0
	Exponential fit (before the inflection)	*f*(*x*) = *a* × exp (*b* × *x*)
3	[Exp] Decaying factor	*b*
4	[Exp] Initial factor*	*a*
5	[Exp] Goodness of fitting	Using RMS of errors
6	[Exp] Curvature	Ʃ |d*V*/d*t*|, normalized
7	[Exp] Skewness of curvature	Skewness of Feature #6
8	[Exp] Area	ʃ *f*(*t*)·d*t*, normalized
	Lineal fit (after the inflection)	*F*(*x*) = *a* × *x* + *b*
9	[Lin] Slope	*a*
10	[Lin] Intercept	*b*
11	[Lin] Goodness of fitting	Deviation, normalized
12	[Lin] Curvature	Ʃ |d*V*/d*t*|, normalized
13	[Lin] Skewness of curvature	Skewness of Feature #12
	Power spectra	
14	SumPower [<64 Hz]*	Summated power between 1 and 64 Hz
15	1st moment [<64 Hz]*	Ʃ (dPower × dFrequency)/Ʃ dPower
16	SumPower [64–256 Hz]	Summated power between 64 and 256 Hz
17	1st moment [64–256 Hz]*	Ʃ (dPower × dFrequency)/Ʃ dPower
18	SumPower [>256 Hz]*	Summated power between 256 and 1024 Hz
19	1st moment [>256 Hz]	Ʃ (dPower × dFrequency)/Ʃ dPower

*Note*: This table summarizes the features extracted from the evoked responses in the perturbed sections that were used to predict preictal periods.

Abbreviation: RMS, root mean square.

* Determined to be the best in signaling the preictal periods in this category.

**FIGURE 2:**
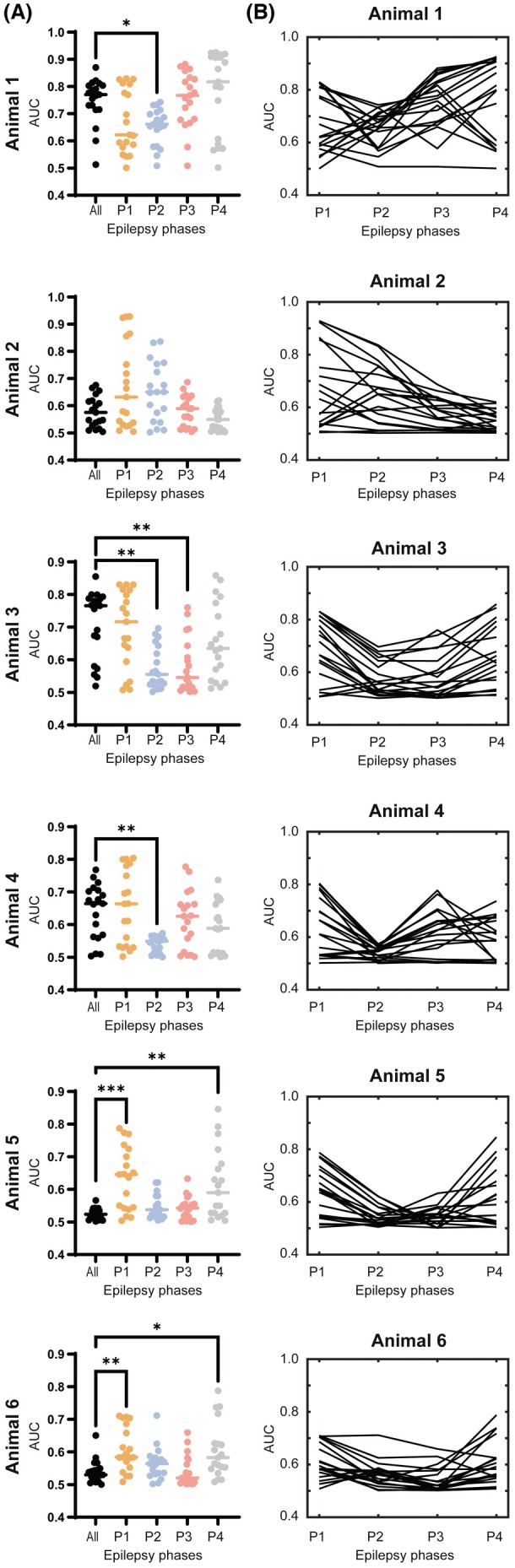
Discriminating interictal and preictal periods using individual perturbation features. (A) Discrimination between the interictal and preictal periods from the whole recording of an animal (all black dots) or the four consecutive epileptic phases (P1–P4, colored dots). Each dot is the result of one of the 19 features from perturbed data (see Table 1). Only Animals 1 and 3 had features from the entire recording (All) with area under the curve (AUC) > .8. Several of the animals had significant differences when analyzing shorter periods versus the entire record. *p < .05, **p < .01, ***p < .001. (B) The AUCs of the 19 features change over the epileptic phases in different animals. Notice every AUC is presented as the greater number between the original one and 1 − AUC; thus, all are normalized over .5.

We next evaluated how well the preictal changes could be discerned from the interictal periods using the candidate features, which could potentially identify the preictal state. The total amount of all 30‐min preictal periods was 14.5% of the animals' recording time. For the interictal periods (59.6% of the recording time), we randomly selected 1800.8 ± 100.2, 30‐min windows that were at least 60 min before any imminent seizure. We compared the 30‐min interictal periods with 348.2 ± 48.9, 30‐min preictal periods per animal (Supplementary S3, odd columns). ROC curves were utilized to discriminate each feature from the preictal versus interictal periods, and we took the AUC to determine how successful the separation was. Perfect separation is an AUC of 1 or 0, and random distribution is .5. When individual features from all preictal and interictal periods across the entire recording were compared, only Animals 1 and 3 had satisfying AUCs (>.8) from their best individual features (Figure 2A, All phases).

Epilepsy is progressive, and the dynamics preceding seizures change with time, especially for the TeNT model,^25^ so we supposed that features and the discriminating power using features would change over time. To validate this, we partitioned each animal's seizure history into four phases with an equal number of seizures and compared the AUCs in each phase to see how the interictal/preictal discrimination varied over time. We found drifts in the features over time, which were sometimes more pronounced than the differences between interictal and preictal periods (Supplementary S4). Importantly, because of the drifts, a good (or bad) discrimination from the whole recorded time could come from falsely comparing a preictal period and an interictal period weeks away. Therefore, we stratified the preictal/interictal analysis into the four phases, keeping the analyses restricted to within each phase (Figure 2A). All AUCs tended to fluctuate with time, suggesting the drifts in features developed along with the progress of epilepsies (Figure 2B); however, we did not discover consistent changes in the AUCs over time that can be utilized for seizure prediction.

Using the timing of preictal and interictal periods from the TeNT animals, we performed the same analysis and comparisons of the 19 features between the “interictal” and “preictal” periods in the control animals. Features are rather symmetrical between the “interictal” and “preictal” periods in the control animals (Supplementary S3, even columns). AUCs of the discriminations between “interictal” and “preictal” periods from the control animals were inspected in the entire recording time or different phases (Supplementary S5A); also, AUCs from the control animals were statistically compared with AUCs from the experimental animals (Supplementary S6). In the total of 24 comparisons, we observed 10 significantly higher AUCs and another eight AUCs that trended higher acquired from the epileptic animals, suggesting the “interictal” and “preictal” periods in the control animals are unlikely to be discriminable.

### 3.3 Create a multivariate seizure‐forecasting model using perturbation features

Although individual features did not reliably discern between interictal and preictal data, a common machine learning approach is to combine them in a multivariate approach for better results. In addition, the data suggested that the responses changed over time during the course of recording, and thus the algorithm should train on shorter time periods rather than mixing data from the entire recording.

**FIGURE 3:**
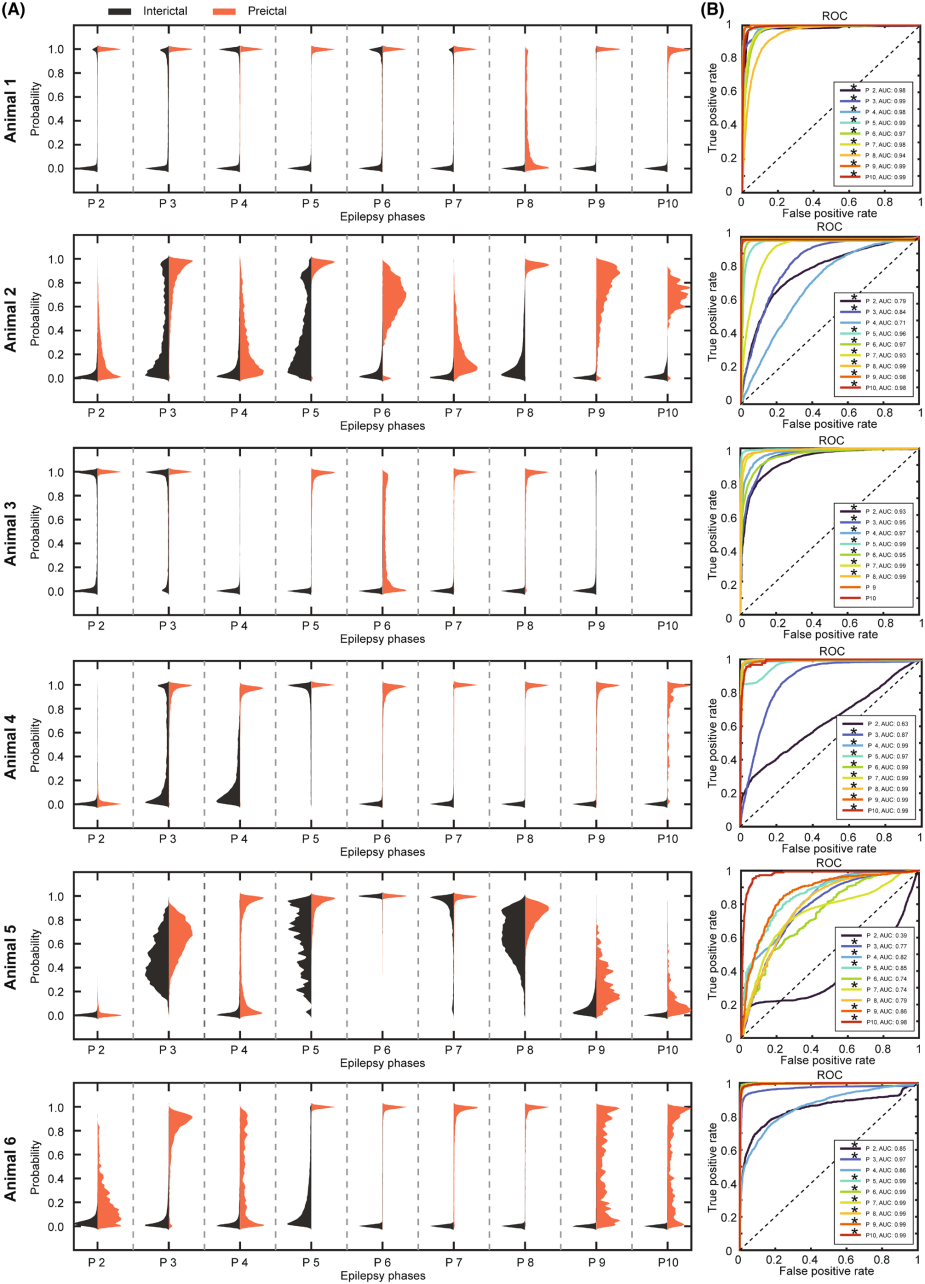
Seizure forecasting using multivariable logistic regression with perturbed features. (A) The entire recording (as the whole epilepsy) of animals is divided into 10 phases; combined violin plots show the values from the interictal (black) and the preictal (red) periods based on the logistic regression models using the five best features from the perturbed sections (indicated in Table 1). (B) Receiver operating characteristics (ROCs) and areas under the curve (AUCs) from phases reflect the successful rates of the prediction; all animals had excellent AUC by the later phases. *p < .05, indicating the difference from the permuted AUCs.

First, we chose five features with the best interictal versus preictal AUCs based on their means from all the partitioned phases (Supplementary S7): summated power within 1–64‐Hz band, summated power > 256 Hz, the initial factor 'a' from the exponential fit, and first moment within the 64–256‐Hz and 1–64‐Hz band (indicated in Table 1 and Supplementary S7). To build a preictal prediction system, we applied a multivariable logistic regression model that rolled with time for each animal individually, using a prospective internal cross‐validation with prospective novel data. To simulate realistic clinical data and account for known changes during the wane and wax of seizures of the models, we split the entire recording into 10 consecutive epileptic phases. At each phase (P), an interictal versus preictal logistic regression model was trained using the five best features, then tested in the subsequent phase (P + 1; Figure 3A). This ensured that each model was tested on novel data and simulated a realistic real‐time implementation in which the algorithm could be updated with prior results at given phases. Overall, the classifier showed good performance in most phases (AUC > .9; Figure 3B), and was significantly different from the permutation analysis (*p < .05, marked Figure 3B) and single feature‐based discrimination. In addition, we noted that performance tended to be better in the later phases. Therefore, we tested the correlation between phase and AUC by fitting them to a linear model in all animals. We found the AUCs are positively modulated by increasing (i.e., later) phase (*p* = .001, *F*_1, 50_ = 12.26, *R* ^2^ = .20). These statistical tests suggest our multivariable prediction system is effective at discriminating preictal from interictal data even at the first phase (AUC ~ .82) and tends to improve thereafter in later phases.

Using the same criteria, we verified the best five features that individually discriminated the “preictal” from the “interictal” periods in the control animals (Supplementary S8) to build similar multivariate logistic regression models to predict the “preictal” periods in the next phase. The average AUCs do not discriminate well (AUC ≈ .5; Figure S5).

### 3.4 Perturbation is required to reveal the preictal features

Next, we evaluated how well our system can predict preictal periods using passive (i.e., unperturbed) sections of the recording. Recall that the protocol alternated between sections with and without perturbations (Figure 1C). Without perturbation, many of the features in Table 1 were not applicable, so we extracted additional features that have been used in similar studies in unperturbed data.^24^ Features were pulled from 10‐s rolling windows (without overlaps) of the unperturbed data (Table 2). We performed the same analysis using the individual features as in the perturbation analysis and aimed to build a five‐feature logistic regression model using the five most promising features. The selections and time ranges of the preictal and interictal periods were the same as what was used for the perturbed data, with a 5‐min delay.

**TABLE 2:** List of features characterized from the unperturbed sections.

	Features	Definitions
	Basic	
1	Variance*	Ʃ (*x*_i_−*x*_m_)^2^/(*n*−1)
2	Curvature*	Ʃ |d*V*/d*t*|, normalized
3	Skewness	Skewness of recording
	Power spectra	
4	SumPower [<64 Hz]*	Summated power between 1 and 64 Hz
5	1st moment [<64 Hz]	Ʃ (dPower*dFrequency)/Ʃ dPower
6	SumPower [64–256 Hz]	Summated power between 64 and 256 Hz
7	1st moment [64–256 Hz]	Ʃ (dPower*dFrequency)/Ʃ dPower
8	SumPower [>256 Hz]	Summated power between 256 and 1024 Hz
9	1st moment [>256 Hz]	Ʃ (dPower*dFrequency)/Ʃ dPower
	Autocorrelation (lag – 1)	
11	Autocorrelation [<64 Hz]*	
12	Autocorrelation [64–256 Hz]*	
13	Autocorrelation [>256 Hz]	

*Note*: This table summarizes the features extracted from the unperturbed sections that were used to predict preictal periods.

* Relatively successful in signaling the preictal periods in this category.

**FIGURE 4:**
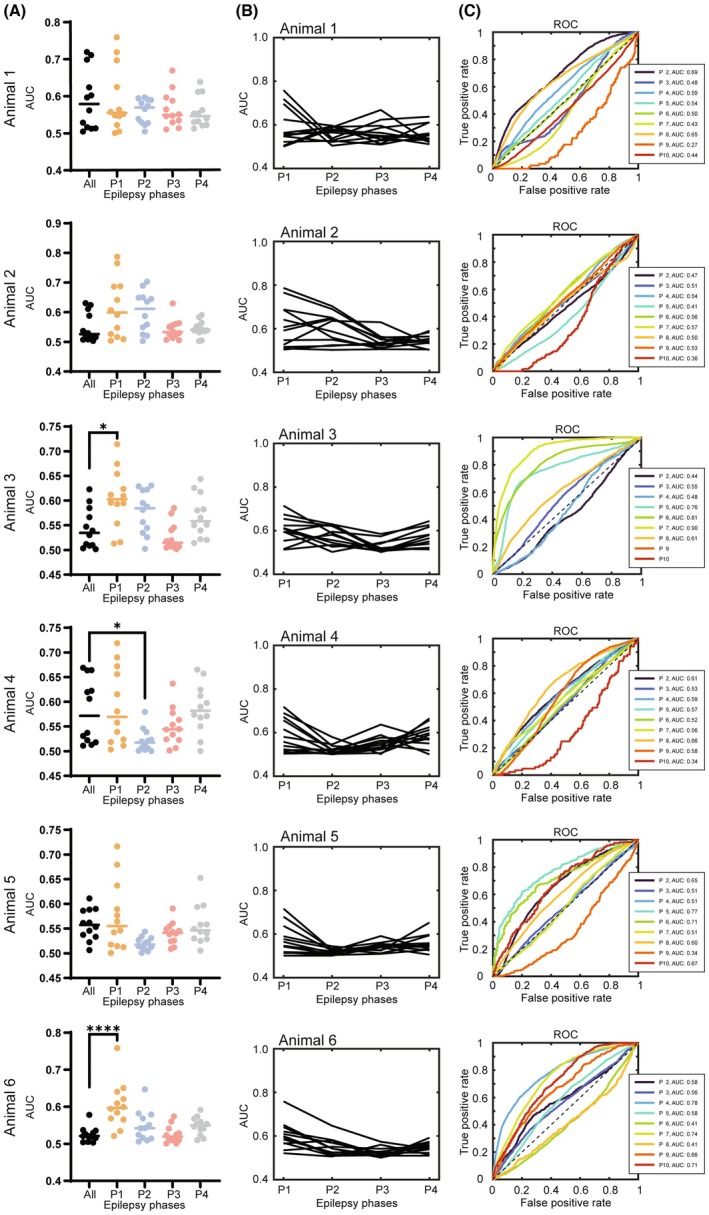
Seizure forecasting using multivariable logistic regression with unperturbed features. (A) Discrimination between the interictal and preictal periods from the entire recording of an animal (all black dots) or from the four consecutive epileptic phases (P1–P4, colored dots) using the 13 features extracted from the unperturbed sections. *p < .05, ****p < .0001. (B) The areas under the curve (AUCs) of the 13 features change over the epileptic phases in different animals. Note that every AUC in panels A and B is presented as the greater number between the original one and 1 − AUC; thus, all are normalized over .5. (C) Receiver operating characteristics (ROCs) and AUCs show that the preictal prediction system using the five successful features from the unperturbed recording is not far from .5.

AUCs from the unperturbed data (Figure 4A) tended to be lower than perturbed data (Figure 2A). We also observed the fluctuation of individual AUCs, but similarly, there were no significant long‐term patterns (Figure 4B). The best five features from the unperturbed sections were identified by the highest means of phasic AUCs along the recording (Supplementary S9); we used them to build multivariable logistic regression models as described before. These passive features were generally unable to distinguish the preictal from interictal periods (Figure 4C). We performed linear regression between AUC and the phase, which was not significant (*p* = .38, *F*_1, 50_ = .79, *R* ^2^ = .02). These results suggest that passive features from the unperturbed data are not effective at distinguishing interictal from preictal data and do not improve in later phases. Note, however, that this analysis only had one feature in common with the perturbed analysis in Figure 2 (summated power within 1–64 Hz).

**FIGURE 5:**
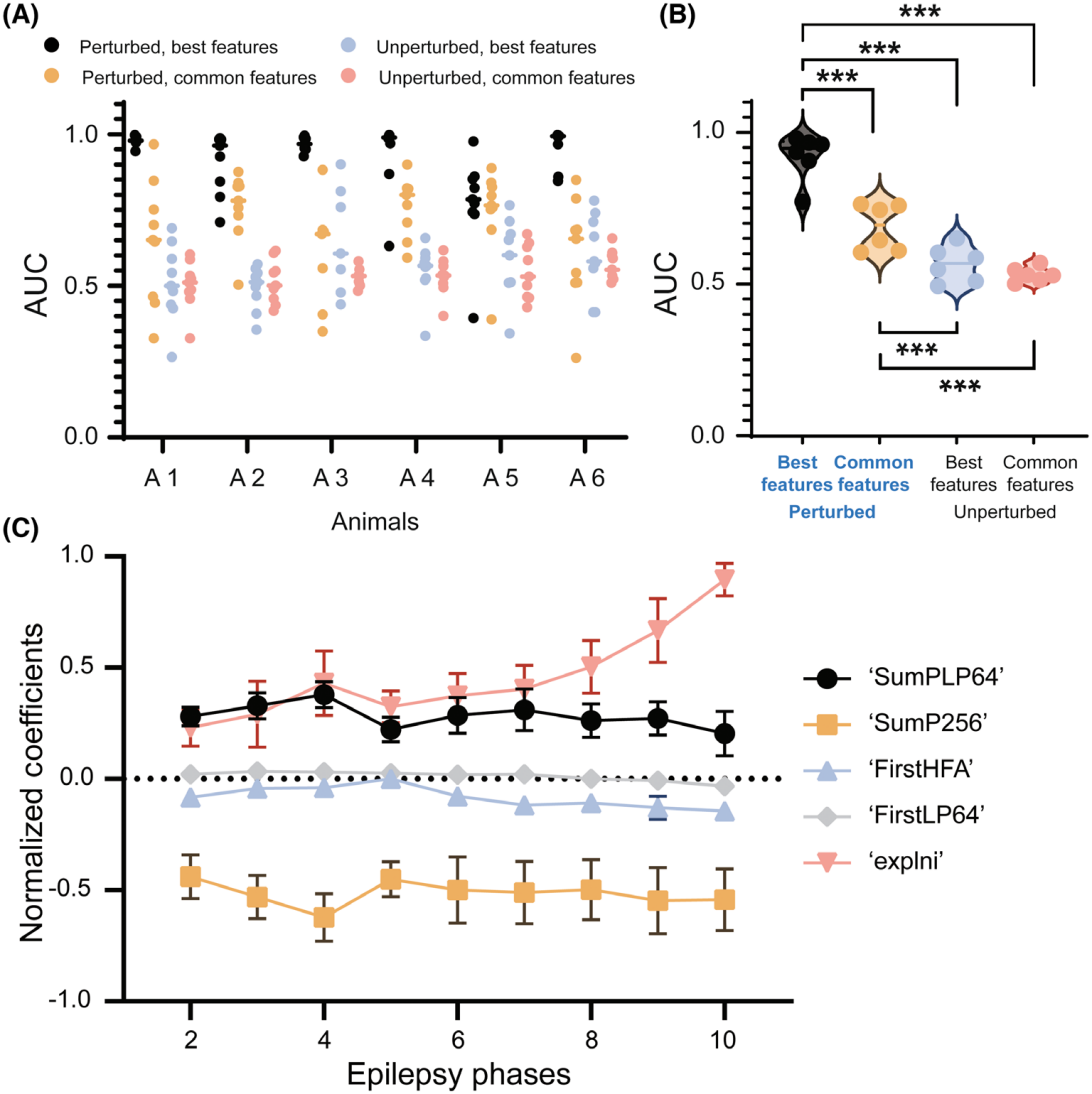
Comparison of seizure forecasting with perturbed versus unperturbed features. (A) A scatterplot summarizes the areas under the curve (AUCs) of the prediction systems in different animals using the best or common features from the perturbed or unperturbed sections. A dot is the AUC of a method in an epileptic phase. (B) The prediction systems using the perturbed features surpass those using the unperturbed features; using the best features grants a higher successful rate than using the common features. Here, the effect of epileptic phases on AUCs is pooled and showed as the averages in individual animals (two‐way analysis of variance, ***p < .001). (C) Normalized coefficients from the logistic regressions suggest the importance of features in discriminating the preictal periods from the interictal periods.

To compare the perturbed and unperturbed data directly, we repeated the analysis using the same features in both data. Here, we chose five features that were not exclusive to the perturbed response: the summated power of 1–64 Hz, 64–256 Hz, and 256–1024 Hz, and the first moment in the 1–64‐Hz and 64–256‐Hz bands. We compared the results of this common‐feature model with those best features from the perturbed and unperturbed sections (Figure 5A). In all animals, when using the same features for analysis, the perturbed features were superior to the unperturbed. In addition, the best feature method performed better than the common‐feature method in both unperturbed and perturbed data (Figure 5B; *p* < .0001, *F*_2.069, 95.19_ = 141.8). In the comparisons, the model using the best features from the perturbed sections is clearly superior in distinguishing the preictal from interictal periods (*p* < .0001). Thus, we can conclude that using specific features from the perturbed sections has superior ability to discriminate between preictal and interictal periods. Finally, the coefficients in the logistic regression models reveal that although common features, such as SumP256 and SumPLP64 (summated power in the >256‐Hz and 1–64‐Hz bands) were consistently important in detecting the preictal state, the perturbation‐specific feature explni (initial factor of the exponential fit) became crucial particularly in the later phases of epilepsy (Figure 5C). First moments in frequency bands 64–256 Hz ("FirstHFA") and 1–64 Hz ("FirstLP64") contributed the least.

## 4 DISCUSSION

A growing body of work demonstrates that actively probing the brain provides real‐time assessment of seizure risk.^17^, ^19^, ^20^, ^23^, ^28^ However, most prior works were limited to trials 1 day long or less and often required manipulation to induce seizures within that brief period. The longest trial to date used implanted devices in humans over the course of a year but did not have true perturbations and had to treat naturally occurring spikes as stimuli to the system.^24^ In the current work, we applied consistent electrical stimuli over several weeks and were able to follow the natural cycling of seizures over time in the TeNT model. These results provide a robust, novel demonstration of the response to stimulation over the course of months that follows the full progression of epileptogenesis in freely moving animals.

Our results show that low‐amplitude, periodic, consistent electrical perturbations to an intrahippocampal TeNT‐induced epilepsy model (*n* = 6 animals) are able to distinguish preictal from interictal states with excellent results (most AUCs > .95). These results were not dominated by any particular feature, because no single feature had strong, consistent performance. Rather, our model required logistic regression using five signal features. Our method of training on available data then testing of prospective data assures these results are not due to overtraining. In contrast, using the same approach with passive EEG biomarkers was not nearly as effective. In an “apples‐to‐apples” comparison, it was clear that the biomarkers from active probing were superior to passive biomarkers, similar to recent results in a different mouse model of epilepsy.^18^ Finally, we also provide results from three control rats that show that the protocol does not trigger false alarms.

One important novel aspect of this work is that it demonstrates the ability of active probing to distinguish the preictal from the interictal state. Prior experimental work with active probing primarily focused on finding changes associated with increased seizure risk^17^, ^19^, ^20^, ^28^ but has not directly tested the ability to forecast seizures. One study that did test long‐term seizure forecasting used epileptic spikes as surrogate perturbations and showed that they were more effective than any prior methods with merely passive biomarkers.^24^ Two recent short‐term studies also showed that active probing was superior to passive EEG.^18^, ^23^ Here, we provide additional corroborating evidence that active probing is superior to passive EEG analysis for identifying the preictal state. Note that our analysis was not designed to optimize the results but was nevertheless able to show via data‐driven analysis that active probing provides superior information to passive EEG.

There were several limitations to our work. First, the TeNT model has frequent seizures that occur in clusters. This raises the possibility that the interictal times might be too short to identify. We defined the preictal period as the 30 min prior to each seizure, and interictal to be greater than 60 min prior to a seizure. We redacted the 30‐min transition period (31–60 min before the seizure) to be conservative (Supplementary S2) and skipped preictal periods that were less than 30 min long. The interictal and preictal periods respectively occupied ~59.6% and ~14.5% of the total recorded time, with another 14.5% redacted. The remaining 11.4% of the time included seizures and seizure clusters that were too frequent to allow distinction between preictal and interictal. Although not included in the final analysis, we also analyzed the perturbed responses during seizure clusters and found them to be highly distinguishable from interictal responses just before the cluster (Supplementary S10), suggesting this method could potentially inform whether the brain has not escaped from the seizure clusters.

Another limitation is that the TeNT model has unique characteristics that are both interesting and challenging. The natural progression of epileptogenesis and seizure remission allows for objective measurement of how seizures progress over time, which is an important physiological question.^25^ However, these changes also make it very difficult to develop a single algorithm that would be effective for the entire period. Our solution to this was to divide the data into phases and allow the algorithm to train on recent data and test on the upcoming phase. However, this rapid progression of epileptogenesis is likely very different from the changes in most human epilepsies. In addition, the waning of seizures in the second half of the recording period is also dissimilar to human epilepsies. Thus, it is unclear how well these results pertain to human epilepsy, particularly in the later stages of seizure waning.

The third limitation is that the recording site (a cortical screw electrode) was not placed in the hippocampus. This meant that the recorded potentials were not necessarily from the cells in the seizure focus and could represent some downstream effects in connected brain regions and/or an attenuated response from the seizure focus. That discrepancy may explain why the responses were not the typical critical slowing responses described in prior work. Finally, our protocol added perturbations quite rapidly (every 3.01 s). This high frequency allows for many robust samples but risks producing long‐term effects that may influence the results. We did not identify changes in the control animals (which received the same stimuli) over time, suggesting the effects were minimal; however, future work would require slower stimulation protocols and/or a direct measurement of the stimulation effect.

## 5 CONCLUSIONS

As wearable and implantable technologies for epilepsy are being developed, the ability to provide patients and caregivers with real‐time information about seizure risk is becoming reality. This work demonstrates that perturbing stimulation provides greater accuracy in identifying imminent seizure than simply analyzing passive EEG signals. We identified a reliable method for distinguishing the preictal from the interictal state in the TeNT model in rats, which adds to the list of epilepsy types in which perturbing stimulation has been successful in assessing seizure risk. These findings in the TeNT model motivate future studies to test and optimize this effect across additional animal models and potentially in humans.

## AUTHOR CONTRIBUTIONS

*Conceptualization:* Wei‐Chih Chang, Mark J. Cook, David B. Grayden, and William C. Stacey *Investigation and data curation:* Warwick Cheung and Alan Lai. *Methodology and analysis:* Wei‐Chih Chang, Jack Lin, Warwick Cheung, Alan Lai, and William C. Stacey. *Visualization:* Wei‐Chih Chang and Jack Lin. *Writing:* Wei‐Chih Chang, Jack Lin, Mark J. Cook, David B. Grayden, and William C. Stacey. *Funding acquisition:* Mark J. Cook and William C. Stacey. *Supervision:* William C. Stacey.

## FUNDING INFORMATION

This work was funded by the National Health and Medical Research Council Project Grant (AppID 1 065 638; M.J.C.), the BioInterfaces Institute (W‐C.C. and W.C.S.), the National Institutes of Health (R01‐NS094399; W.C.S.), the Melbourne International Fee Remission Scholarship (W.C.), the Melbourne International Research Scholarship (W.C.), and Michigan Medicine (Robbins Family Research Fund and Lucas Family Research Fund; W‐C.C. and W.C.S.).

## CONFLICT OF INTEREST STATEMENT

None of the authors has any conflict of interest to disclose. We confirm that we have read the Journal's position on issues involved in ethical publication and affirm that this report is consistent with those guidelines.

## Supporting information


DATA S1.

## Data Availability

All features in the study have been uploaded to https://deepblue.lib.umich.edu/data/anonymous_link/show/1d0e09b53d4a6ef0f94d41d3fc3fb080cb9a5255741bfa59154232393b2ebd90?locale=en. The original recordings are available from the corresponding author upon request.
