# Supplementary material for: Perturbation‐induced responses improved seizure forecasting in epileptic rats
Source: Epilepsia. 2026 Mar 13;67(6):3158–70. doi: 10.1002/epi.70196 (PMC13285227; doi:10.1002/epi.70196)
Supplement: Supplementary file 1 — DATA S1. [file EPI-67-3158-s001.docx]

­­Title:

**Perturbation-induced responses improved seizure forecasting in epileptic rats**

Authors:

**Chang W.-C., Lin J., Cheung W., Lai A., Cook M.J., Grayden D.B., Stacey W.C.**

**Supplementary Information**

**Supplementary 1.**


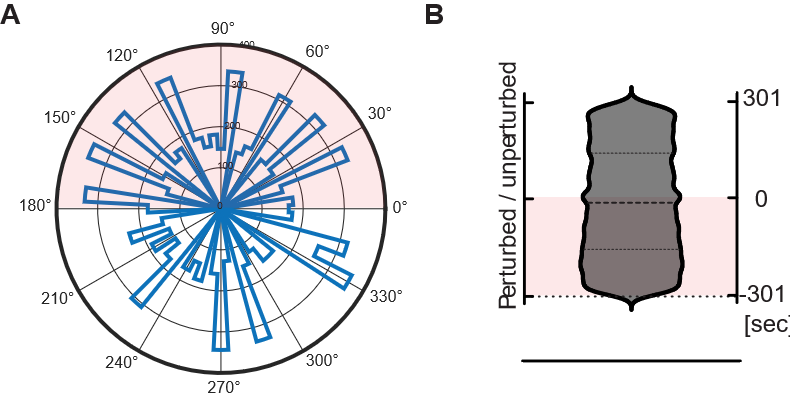


**Supplementary 1, Seizure timing and perturbation phases.** (A) The polar histogram demonstrates the temporal relationship between seizure onsets and perturbation; all seizures in all animals are included. The perturbed phase is from 180° through 90° to 0° (red shade), and the unperturbed phase is from 0° through 270° to 180°. The radius represents the incidence of seizures. (B) The violin plot presents the incidences of seizures in the perturbed and unperturbed phases. The distribution of seizure onsets was tested as a uniform distribution (*p*<0.0001), suggesting the seizures evenly started from any moment, independent of the perturbations. However, there is a slight skew, 0.09, toward the perturbed sections.

**Supplementary 2.**


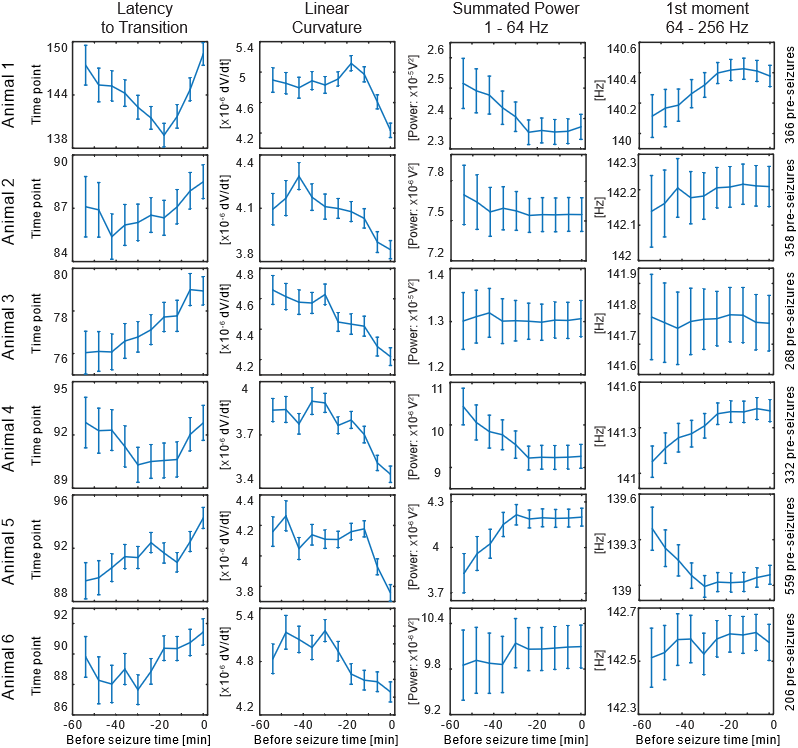


**Supplementary 2, Preictal changes of the selected features.** This figure reveals the fluctuations of four selected features (in columns) in the preictal 60 minutes from the six epileptic animals (in rows). The numbers of preictal periods making the plots are given at the right end of each row by animals. We looked for changes in values or in the trends of the features before seizures and expected them to be good indicators for the proximity of seizures. Notice that a successful feature in one animal can be unsuccessful in another. When a feature may be useful in signaling seizures, the changes usually happen within the preictal 30-minute periods or around the 30^th^ minute preictally.

**Supplementary 3.**


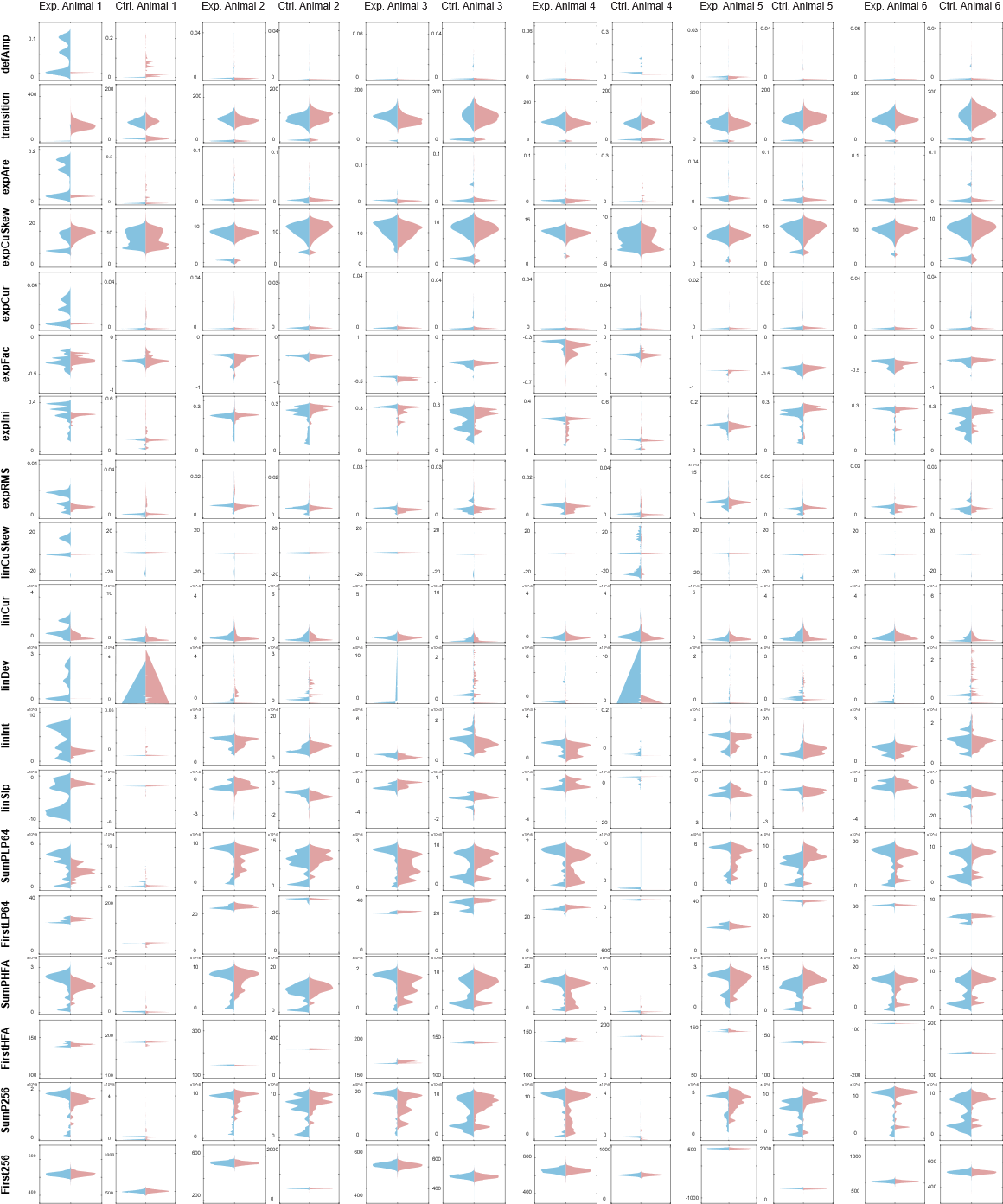


**Supplementary 3, Violin plots of the 19 features from all interictal and preictal periods of the experimental and control animals used in this study.** Features are presented in the rows, and animals in columns when the data from an experimental animal is followed by its paired control. Features from the interictal periods are given in blue, and features from the preictal periods are red. Notice the numbers were normalized within either the interictal period groups or the preictal period groups.

**Supplementary 4.**


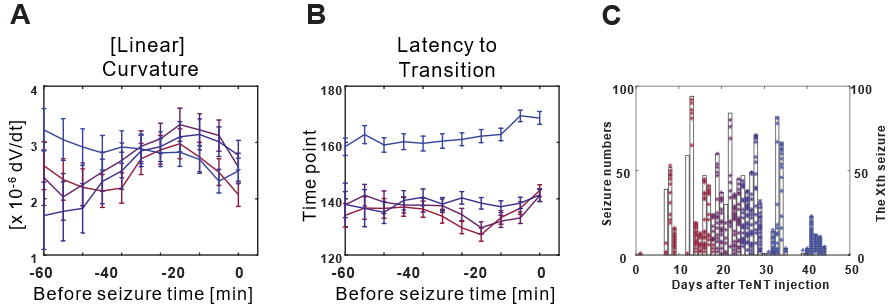


**Supplementary 4, Examples of the long-term (epileptogenic) effect on features.** (A) The curvatures from the linear component, as one of 19 features, remained in the same range and a similar trend throughout the lifetime of this animal. (B) In the same animal, latency to the transition point, as another one of 19 features, from the last quartile of epilepsy (the whole recording) had a significant deviation from the first three quartiles. (C) This plot points out the timing of seizures in the days of epilepsy. The epileptic phases were divided based on the number of analyzed seizures. Seizures in the first quartile are marked in red and blue for seizures in the last quartile; seizures in the 2^nd^ and the 3^rd^ quartiles are coded in purple with different red and blue gradients. The colors in (A) and (B) correspond to (C).

**Supplementary 5.**


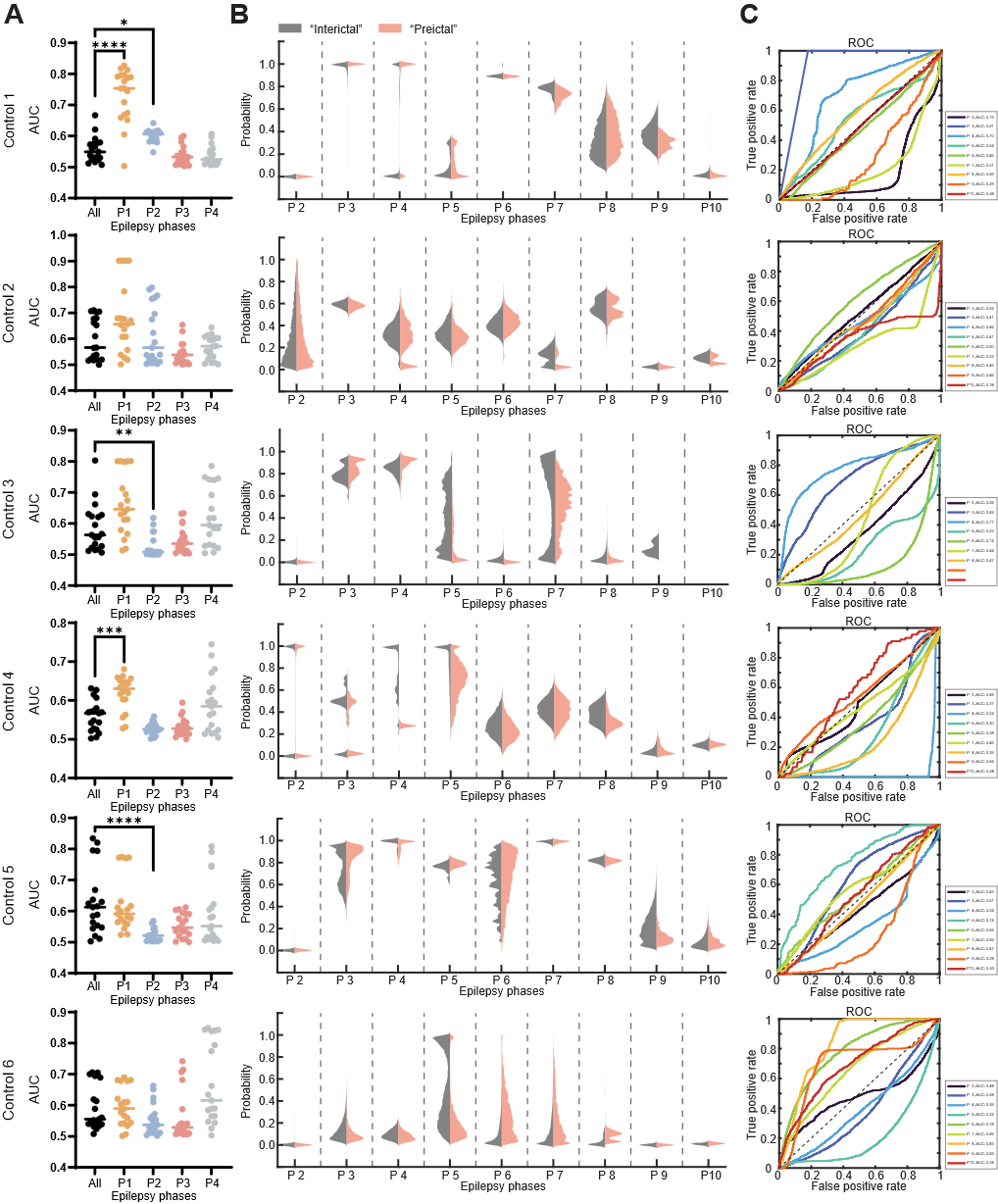


**Supplementary 5, Discrimination between the “interictal” and “preictal” periods in the control animals.** The interictal and preictal timing were determined by the control animals’ paired epileptic rats; the 19 perturbed features from the perturbed sections of the interictal/preictal periods were regrouped as from the whole recording of an animal (All, all black dots) or from the four progressing epileptic phases (P1 to P4, colored dots). *, *P*<0.05; **, *p*<0.01; ***, *p*<0.001, and ****, <0.0001.

**Supplementary 6.**


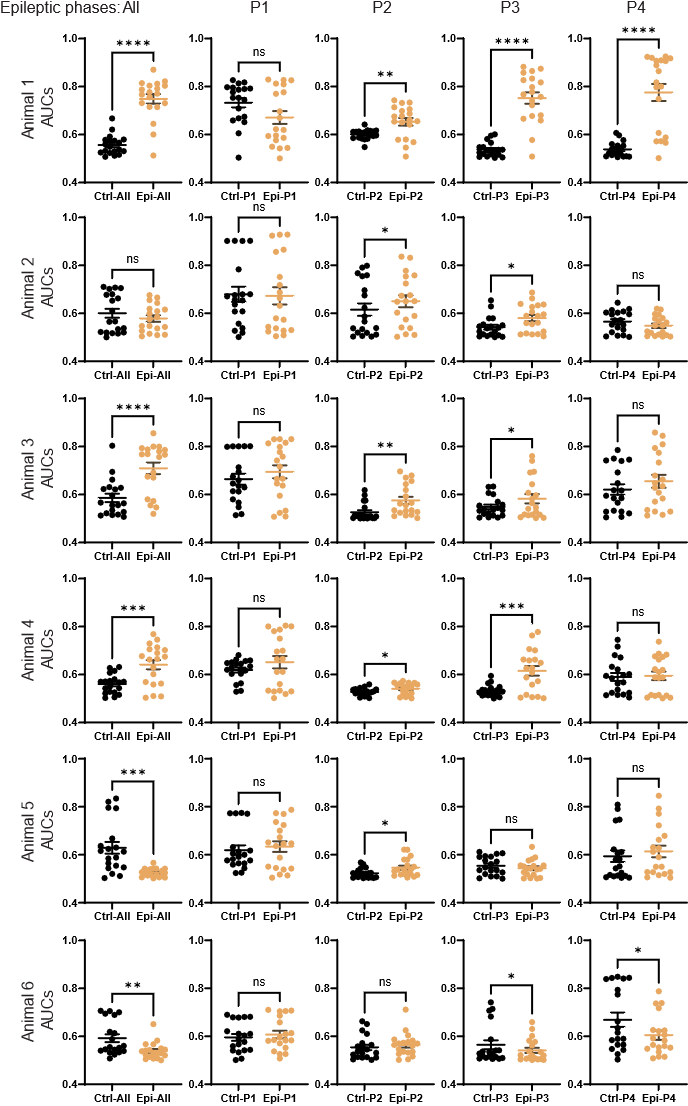


**Supplementary 6, AUCs from the control and experimental animals from the whole or different phases.** Discriminating AUCs of 19 features from the control (black dots) and the experimental animals (yellow dots). *, *P*<0.05; **, *p*<0.01; ***, *p*<0.001, and ****, <0.0001.

**Supplementary 7.**


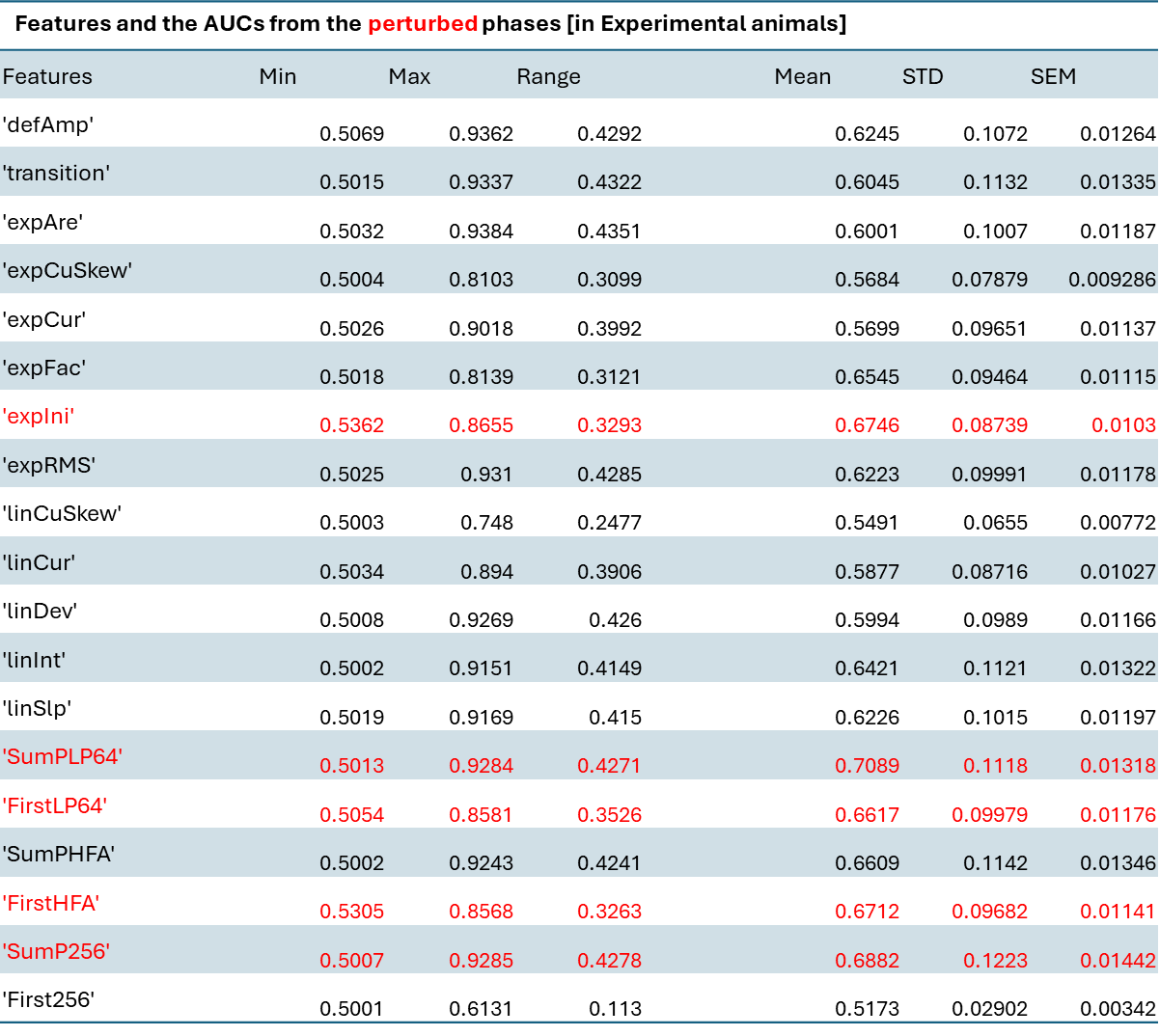


**Supplementary 7, AUCs of the 19 perturbed features in experimental animals.** The best five features were determined by the highest mean (in red).

**Supplementary 8.**


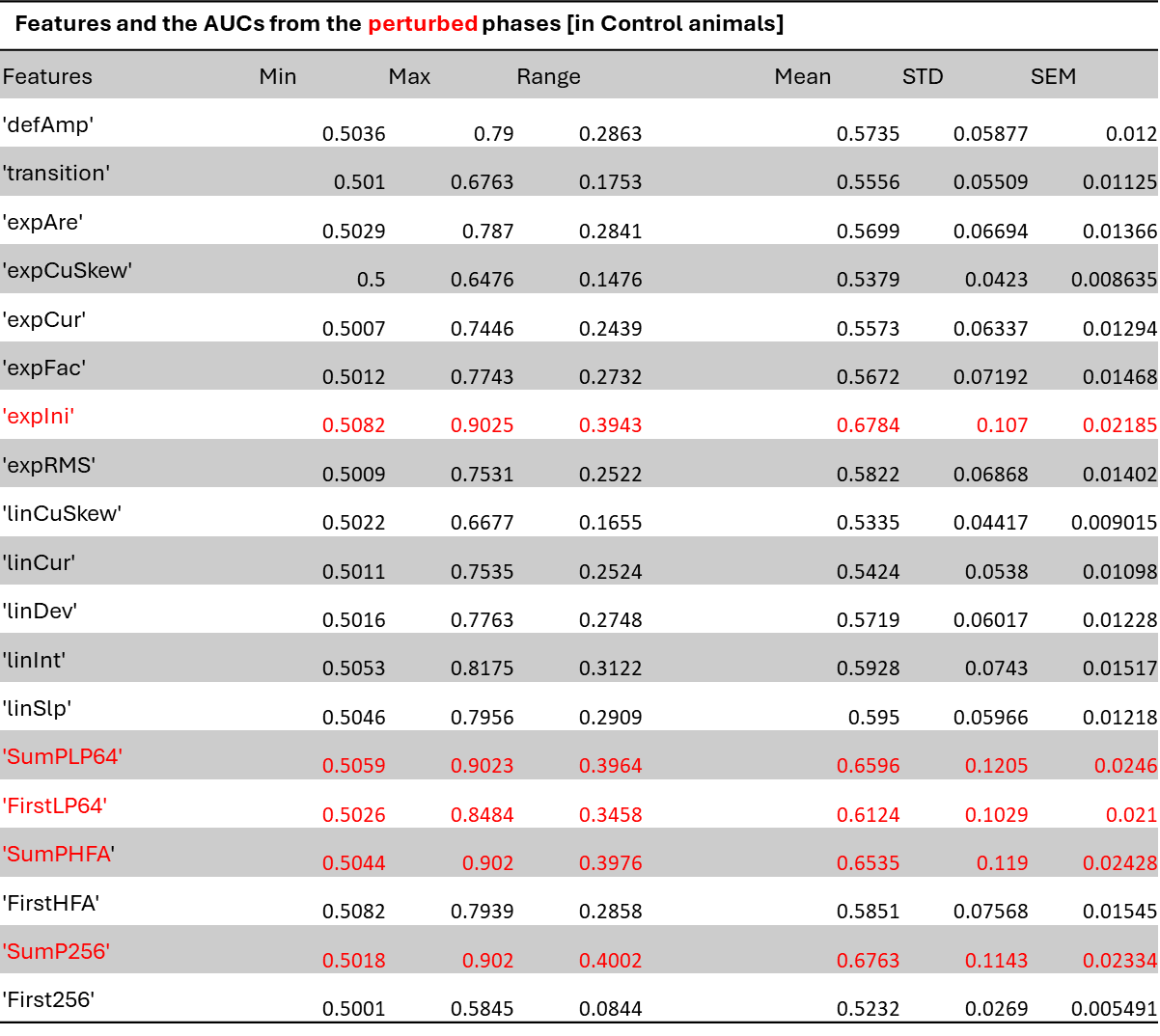


**Supplementary 8, AUCs of the 19 perturbed features in control animals.** The best five features were determined by the highest mean (in red).

**Supplementary 9.**


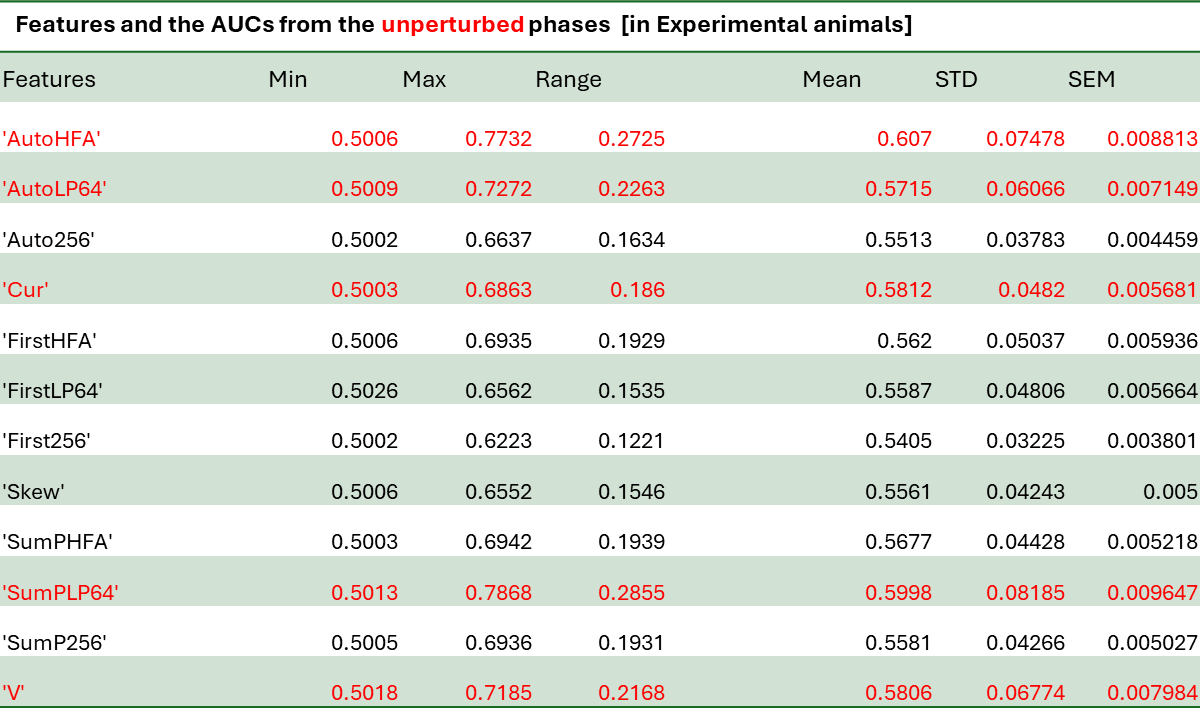


**Supplementary 9, AUCs of the 13 unperturbed features in experimental animals.** The best five features were determined by the highest mean (in red).

**Supplementary 10.**


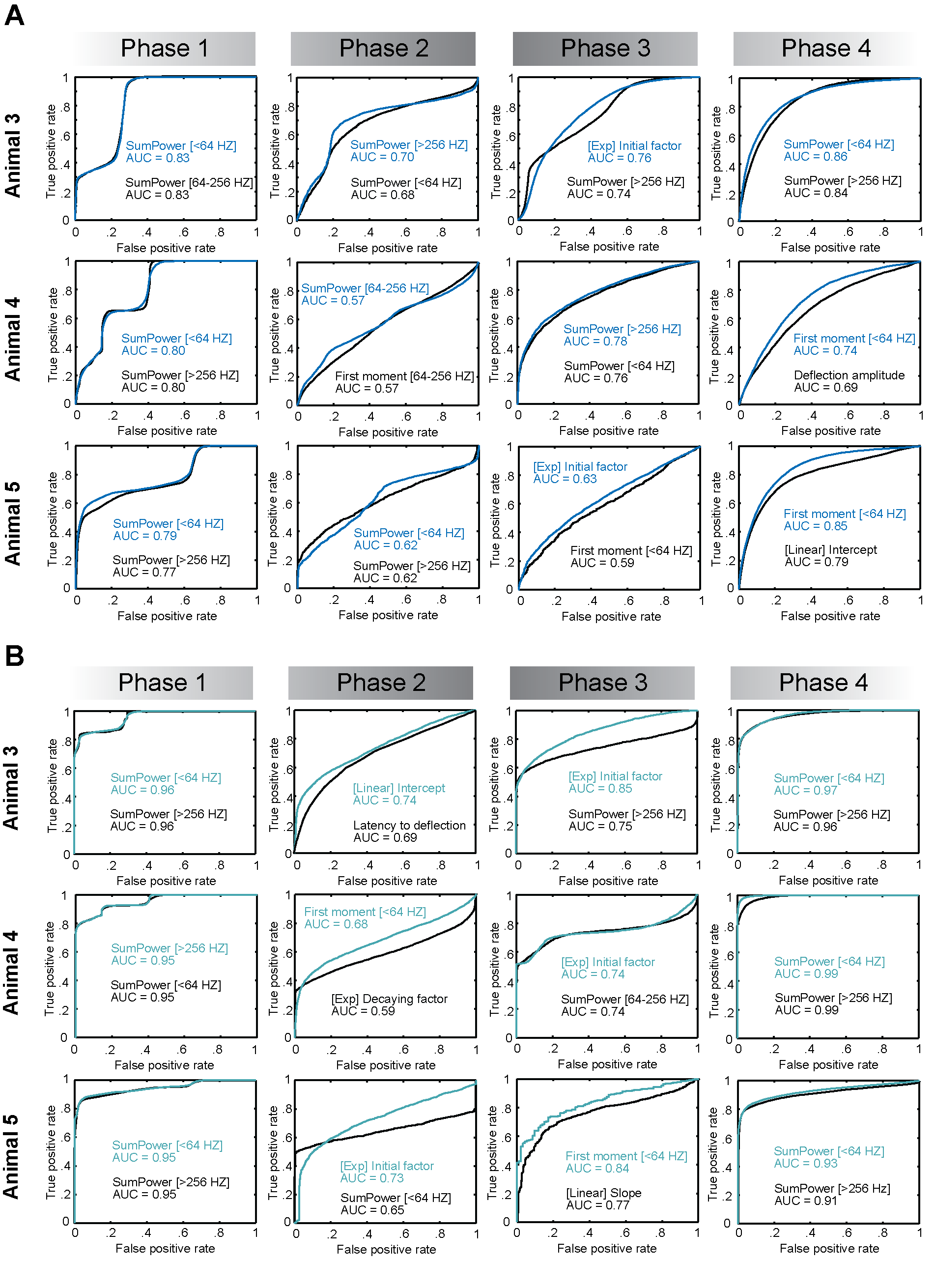


**Supplementary 10. Distinguished preictal states in seizure clusters.** (A) From the same dataset of Figure 2A, ROCs and AUCs of the two best perturbed features are shown here. The discrimination, which is only poor to moderate, was found between the preictal periods (30 minutes before any imminent seizure) and the 30-minute interictal periods (>60 minutes away from an imminent seizure). (B) However, the ROCs and AUCs are improved when the same features were used to discriminate the preictal 10-minute periods within seizure clusters from the interictal periods (>60 minutes away from an imminent seizure).

**Materials and Methods in Details**

Tetanus toxin-induced chronic model of epilepsy and the in vivo experiment

The animal study was approved by the St. Vincent’s Hospital (Melbourne) Animal Ethics Committee and performed in accordance with the Australian Code for the Care and Use of Animals for Scientific Purpose, 8^th^ Edition (2013). Adult male Sprague-Dawley rats were acquired from the Animal Resources Centre (WA, Australia). Six rats received an intrahippocampal injection of 30 ng of tetanus toxin (TeNT, in 300 nl of phosphate buffer saline, PBS); three rats received 300 nl of PBS only. After TeNT or PBS injection, four recording epidural screw electrodes (1.524 mm, E363/20/SPC, Plastic One Inc., VA, USA) were implanted at AP -1.2 or -6.8 mm while ML 3.0 or -3.0 mm respectively (Figure 1A); the reference was above the cerebellum. Electrodes were connected to an off-the-shelf EEG acquisition system (Grael, Compumedics Ltd., VIC, Australia). Animals were transferred back to their single-house cages with 16/8 hours of light/dark cycles and free access to food and water.

From Day 1 (2 days after the surgeries), all nine animals started to receive electrical perturbations (stimuli). All stimuli were controlled/delivered by a stimulator (neuroBi (Slater et al., 2015)) through electrodes 3 and 4. The electrical perturbation protocol is shown in Figure 1B: every stimulus comprises a -1.2±0.024 mA, 500 µs long negative pulse and a 1.2±0.024 mA, 500 µs positive pulse separated by a 20-µs gap (amplitude = 0 mA). Stimuli were added every 3.01 seconds for 301 seconds (100 pulses), followed by 301 seconds without stimuli (Figure 1C). This on/off cycling continued unabated every day for 23 hours each day (allowing a ~1 hour pause for maintenance) for the entire multi-week experiment. The sampling rate of data was 2048 Hz. The impedance of the electrodes was checked daily. There was no significant difference between the electrodes’ initial impedance (3532.5±49.14 Ω), and the impedance increased ~48.56±5.27 Ω each day.

Power Calculation

We estimated the number of animals and data based upon signal characteristics. We were first concerned if the preictal periods are different from the interictal periods in individual animals. For a 1 µV mean and standard deviation difference between the interictal and preictal periods, 36 interictal and preictal periods are needed to determine the *p* = 0.01 significance at a statistical power of 95%. There are up to 30 seizures expected to manifest a day in this model (Jefferys J and Walker M, Tetanus toxin model of focal epilepsy. In: A Pitkanen, PA Schwartzkroin, SL Moshe, editors, Models of seizures and epilepsy 2006). Thus, we can anticipate the interictal periods and preictal periods to be distinguishable every 2 days for a single animal. Given six animals and recordings for over 2 months, the design was considered well powered.

Data analysis

Data analysis was performed in Matlab (MathWorks).

Seizure detection

Seizures were automatically detected, triggered by instantaneous power (13-48 Hz) of the recording, using previously validated methods (Crisp et al., 2020). We performed manual seizure reviewing by two physiologists: ~1.25% of the detections were determined to be false positives and were removed; in addition, the timing of <5% seizure onsets and offsets was adjusted based on visual interpretation. Only seizures with lengths over 10 seconds were used in this study. For each seizure, we determined the phase it occurred within the stimulation cycling: the stimulation phase from 0 to 180 degrees and ‘off’ phase from 181 to 360 degrees (Supplementary 1).

Detecting the stimulating artifacts

The electrical perturbations were given in the left hemisphere, producing distinct, high amplitude stimulation artifacts on Electrode 4 that we used to detect the stimuli using the *findpeaks* function in Matlab. We set a refractory period of 1 s after each detection to ensure each stimulus was only detected once. We validated all perturbations by measuring the number and intervals between the stimuli, which should all be 3.01 s within a train and 301 s between trains. We evaluated each evoked-response individually, setting the time of the detected stimulating artifact (peak) as t=0 ms.

Feature extraction from the perturbation-evoked responses

Our analysis only included interictal data. We thus redacted all responses during seizures and for 10 seconds after each seizure was completed. In this study, we used the 1^st^ to the 1024^th^ data points (thus 500 milliseconds long) after each stimulating artifact as the perturbation-evoked response. To eliminate any baseline DC shift before each stimulus (Figure 1C), every evoked response was offset by the prestimulus baseline taken by averaging from t=-4.9 to t=-29.3 ms (the -10 to -60 data points) before each stimulating artifact. Evoked responses in a 10 s sliding window (no overlaps) were averaged to improve the signal-to-noise ratio. Given the inter-stimulus interval of 3.01 seconds and the on/off epochs every 5 minutes, an average contains 0 to 3 evoked responses.

The evoked responses from Electrode 4 contained a fast-decaying component followed by a slow-decaying component, which typically reversed slope from negative to positive to make a “bump” in the voltage tracing. In some examples, there was a transition where the slope became less negative but the sign of the slope did not change, which was common in the control animals. We noted that this transition occurred primarily in the first 100 ms of the recording. To separate the fast and slow components and account for the two cases (with and without a bump), we found the transition point where the absolute value of the slope (|dV/dt|) is closest to zero within the first 150 data points (until t=73.2 ms) of an evoked response (Figure 1F). We calculated the time (latency) and amplitude of the voltage at this transition point used as features for analysis.

The first, fast-decaying component, from the first data point to the transition point, was fitted to an *exponential model 1* [ f(x) = a*exp(b*x) ] in Matlab. The slow component, from transition point to the 1000^th^ data point (t=488.3 ms), was fit to a generalized linear regression model [ f(x) = a*x + b ] using ‘glmfit’ in Matlab. The fitted parameters were used as signal features for analysis. We also extracted other features of each component: the curvature as the sum of absolute values of dV/dt normalized by the duration of the signal and the skewness of dV/dt of each component.

The summated power and the first moment of frequencies were acquired from the complete evoked responses (i.e. fast and slow components combined). We extracted recordings from the 1033^rd^ to the 10^th^ data points before perturbation as the background (t=-504.4 to -4.4 ms, relative to the stimulus at time 0), and the 1^st^ to the 1024^th^ data points after the perturbation as the evoked responses (from t=0 to 500 ms). We calculated power spectra on both data segments using Matlab, and the background power spectrum was deducted from the power spectrum of the evoked response. Each spectrum was subdivided into 1-64 Hz, 65-256 Hz, and >256 Hz frequency bands, and the summated power (∑ dPower/dt) was found individually; the first moment of frequency was acquired by ∑ (dPower/dt*dFrequency/dt) / ∑ dPower/dt in each band.

Perturbation-evoked responses from the control animals

A similar analysis was performed in three control animals, which were subjected to an identical stimulating protocol but did not have any seizures. We used the same methods to extract features. Each control animal was paired with two of the experimental animals; the timing for the control ‘preictal’ data was determined by using the corresponding times from the matched experimental animal, referenced to the day of injection. The assured time of day was matched between experimental and control subjects.

Feature extractions from unperturbed phases

An unperturbed phase was defined between 3.01 s after the last pulse of a stimulating train and 10 s before the next stimulating train. Instead of using discrete evoked responses for the perturbed phase, we analyzed continuous recordings in 10 s rolling windows (no overlaps) for the unperturbed phases. Data sections <10 s were discarded after data partition. Every 10 s data section was pretreated by *detrend* and *filter* (IIR filter, order: 2, notch: 49-51) to remove DC shift and mains (50 Hz) noise. Variances, curvature, skewness, and the features regarding power spectra were calculated in the same fashion as the perturbed phases. Autocorrelation (lag-1) is a feature previously used to evaluate potential critical slowing down in unperturbed EEG (Maturana et al., 2020). The continuous data were band-pass filtered at 1-64 Hz (IIR filter, order: 70), 65-256 Hz (FIR filter, order: 70), and >256 Hz (FIR filter, order: 70), and the coefficient between the current epoch and the previous epoch was found for the feature, autocorrelation (lag-1). Note that autocorrelation was not necessary in the perturbed epochs because the stimuli allowed explicit testing of the stimulus response; in addition, the baseline during the perturbed periods had a trend that made autocorrelation complicated (see Fig. 1F).

Discrimination between the interictal and preictal periods

A preictal period was defined as the 0-30 minutes before every seizure onset. If a seizure occurred so quickly after a previous one that the preictal period was shorter than 30 minutes, that seizure and its preictal data were excluded from analysis. Interictal periods were any 30-minute segments greater than 60 minutes before the nearest seizure onset: for any longer interictal epoch lasting *l* minutes, we generated *n* 30-minute segments, where *n* is the integer rounded down from *l/*30. We randomized the start times of each interictal period by using a uniform distribution across the full length of *l* using Matlab’s *rand* function; if a start did not lead to a 30-minute interictal period, another start was reelected.

The discrimination between preictal and interictal periods was evaluated for each perturbed feature individually by using Matlab’s *perfcurve* function to generate the receiver operating characteristic (ROC) curve and its area under the curve (AUC). We also divided every TeNT animal’s lifetime into four progressive phases with equal seizure incidence per phase and discriminated features from preictal and interictal periods in every phase, respectively. In the same manner, we evaluated how well the unperturbed features distinguished interictal from preictal periods.

In order to create an animal-specific seizure prediction algorithm, we select the top five perturbed features that individually best separate preictal from interictal periods as described above. Each animal’s timeline is then split into 10 equally segmented, continuous phases without overlaps. The first phase started from the first detected seizure with the last phase ending at the last detected seizure. Each of the ten phase lengths varies between animals from 4 to 6 days. To create our time-rolling preictal detection system, we fit a multivariable logistic regression using Matlab’s *mnrfit*function at each phase (P) classifying preictal from interictal perturbed features described above.  Preictal detection is then tested on the data from the subsequent phase (P+1). The test performances were evaluated by calculating the AUCs of the ROC curves. By performing this process iteratively, for each rat we generate up to nine models (*i.e.,* one from each phase) that were each tested on their respective subsequent phases. A model was only fitted if there were at least 50 data points from the interictal and preictal periods, respectively. As a result, prediction performance at a given phase P+1 was only evaluated when there was sufficient data to generate the model from the previous phase P. Coefficients in the logistic regression models were extracted to reveal the relative importance of features; because the magnitude ranges of the coefficients are different in animals, the coefficients were normalized for presenting. In the permutation test, a control AUC was created by shuffling datapoints in every epilepsy phase of every animal, 500 times; significance is determined by *p*<0.05. Furthermore, we also performed this time-rolling preictal detection system using the top five unperturbed features in the same manner.

Statistics

GraphPad Prism version 10 was used; numbers in the text are given as mean ± standard error of mean in this study. The test results are given as the probability (p) for the F-statistic values. Significance is determined when *p*<0.05 unless otherwise noted. The normality of AUCs from animals, interictal/preictal periods, stimulating sections, or using different features was tested by the Kolmogorov-Smirnov test. 1-way or 2-way ANOVA, either parametric or non-parametric settings, were used to compare between groups. We tested correlations and fit linear regressions to the multivariable AUCs from the progressive phases to demonstrate whether our preictal detection system improved along with the animals’ lifetime.
